# Depression and risk of gastro-oesophageal reflux disease (GERD): results from the UK Biobank study

**DOI:** 10.1186/s12876-025-04591-7

**Published:** 2026-01-08

**Authors:** Julia Reizner, Dennis Freuer, Timo Schmitz, Jakob Linseisen, Christa Meisinger

**Affiliations:** Epidemiology, Medical Faculty, University of Augsburg, University Hospital of Augsburg, Stenglinstr. 2, Augsburg, 86156 Germany

**Keywords:** Depression, Gastro-oesophageal reflux disease (GERD), UK Biobank, Age interaction

## Abstract

**Background:**

This study investigated the association between depression and the incidence of gastro-oesophageal reflux disease (GERD) and examined whether the association interacts with age. The analysis was based on 457,958 participants aged 37–73 years from the UK-Biobank prospective cohort study.

**Methods:**

The baseline examination started 2006 and the participants were followed up until 2019–2023 (median follow-up time 13.52 years [interquartile range12.62–14.27]). Depression at baseline and incident GERD at follow-up were defined through sources of the British health system (ICD-codes) and self-report. Multivariable adjusted Cox regression models were used for analysis. Formal tests for interaction with sex and age were conducted.

**Results:**

Participants who developed GERD during follow-up were characterized by an unhealthier lifestyle and more comorbidities than individuals without GERD. In multivariable analysis, depression was associated with incident GERD (Hazard ratio 1.51 [1.46,1.55]; *P* < 0.001). The association decreased with increasing age. There was no interaction with sex.

**Conclusion:**

Depression and its psycho-physiological consequences may be associated with the development of GERD, in particular in middle-aged people. Consequently, increased attention of the treating physicians regarding an increased risk of GERD in depressed persons is important.

**Supplementary Information:**

The online version contains supplementary material available at 10.1186/s12876-025-04591-7.

## Introduction

Gastro-oesophageal reflux disease (GERD) is a common medical condition. In 2020 around 10–25% of the Western population were affected by the disease [1]. GERD is caused by the lower oesophageal sphincter muscle not closing properly resulting in stomach acid and contents to flow back into the oesophagus. In the long term, stomach acid damages the oesophageal mucosa and can lead to conditions such as Barrett’s oesophagus, oesophagitis, ulcers, open wounds or even cancerous lesions [2]. Symptoms of GERD include heartburn, acid reflux, dysphagia (difficulty swallowing), and chronic cough [2]. Typical risk factors for GERD include rising age (particularly from age 50), obesity, unhealthy lifestyle habits, and the use of non-steroidal anti-inflammatory drugs like acetyl salicylic acid [1, 3]. Mental illnesses are frequently associated with common physical diseases including gastrointestinal diseases [4, 5]; one of the most common mental illness, depression, was suggested as a further risk factor for GERD [6]. In 2018–2020 the prevalence of depression in the European population aged 15 years and older was 6.5% with incidence rates continuing to rise [7–10]. Depression can affect the digestive tract in multiple ways. In addition to overeating and malnutrition, dysregulation of the autonomic nervous system and an increase in inflammatory reactions in the context of existing depression [4, 11], antidepressant medication, mostly selective serotonin reuptake inhibitors (SSRI), can put a strain on the digestive tract [12].

Most previous observational studies have examined the prevalence of depressive symptoms (assessed by validated questionnaires) in people with GERD. These results were summarized in a recent systematic review and meta-analysis [13]. However, so far, there are very few cohort studies to date that investigated the incidence of GERD in persons with depression [14, 15]. A study by Kim et al. using a nested case-control design based on a national sample cohort suggested that individuals with depression may have an increased risk of developing GERD [15]. Due to the still scarce scientific knowledge on this topic, the present study aimed to investigate whether adults from the general population suffering from depression have an increased risk to develop GERD and whether there are sex- and age-differences.

## Methods

### Study population

Data for this study was obtained from the UK Biobank, a comprehensive prospective cohort study. The study population of the UK Biobank comprises 502,151 participants from age 37 to age 73 at baseline examination, and that took place between 2006 and 2010. Participants were followed up until 2019 and 2023 (median follow-up time 13.52 years, interquartile range (IQR) 12.62–14.27). Follow-ups were carried out up to four times. Participant information was regularly updated on hospital admission data, primary care data and death registry data.

Data of all study participants was pseudonymized, and participants gave written informed consent. UK Biobank data collection was ethically approved by the North West Multi-Centre Research Ethics Committee (MREC) [16]. For the present study, we obtained authorisation from the UK Biobank access management and followed their ethical guidelines [17].

### Data collection

In this prospective analysis, there were 465,465 study participants without a diagnosis of GERD at baseline. After exclusion of participants who withdrew their consent (*n* = 18) and 7489 participants due to missing values in some of the covariables, 457,958 participants (*n* = 413,247 without GERD and *n* = 44,711 with incident GERD at follow-up) were included in the analysis. An overview of the study sample was provided as a flowchart (Supplementary figure S1).

At baseline examination, socio-demographic data, including information on education and income, and data on comorbidities such as asthma and chronic obstructive pulmonary disease (COPD) were collected. Furthermore, information on lifestyle data like alcohol consumption, smoking status, and physical activity was gathered [18].

### Exposure

Depression at baseline was defined using the UK Biobank International Classification of Diseases (ICD)-10 code F32 (depressive episodes); cases of recurrent or chronic depression (ICD-10 code F33) were not separately included, as these are already captured under F32. This variable was primarily derived from hospital inpatient records, with additional data from primary care records and self-reported diagnoses. Incident cases of depression occurring after baseline were not assessed. The distribution of baseline cases across data sources is shown in Supplementary figure S2.

### Outcome

The endpoint variable of this study was incident GERD, which was defined through the ICD-10 code K2. Outcome data were obtained from hospital admission data, primary care data, death registry data, self-reports and other sources. Detailed information on available data fields and their sources is publicly accessible via the UK Biobank Data Showcase [19] and is shown in Supplementary figure S3.

### Statistical analysis

Categorical variables were given as absolute frequencies with percentages, and the continuous variable is presented as median and interquartile IQR. To examine group differences between participants who did not develop and those who developed GERD during follow-up. Chi-square tests for categorical variables were performed. For the continuous variable age, a nonparametric test (Mann-Whitney U test) was calculated to assess differences.

### Survival time analysis

Cumulative incidence graphs were created, and log-rank tests were performed to test whether or not the curves differ significantly between persons with and without depression.

### Cox proportional hazards analyses

Follow-up days between baseline examination and the diagnosis of GERD or the date of censoring were calculated. To examine the association between depression and the diagnosis of GERD over time, multivariable Cox regression models were calculated.

Selection of covariables was performed using a directed acyclic graph (DAG) (see Supplementary figure S4). The DAG was constructed using the Dagitty web application (version 3.1) based on a literature review [20]. The final model was adjusted for sex, age, smoking, alcohol consumption, physical activity, COPD, asthma, income, and education. For each variable in the final model, the absence of multicollinearity was ensured by calculating the covariate-specific generalized variance-inflation factor (GVIF). The proportional hazard assumption was checked graphically through Kaplan-Meier plots. For this purpose, the continuous variable age was categorized. The assumption was found to be satisfied for all variables. To improve model fit and account for potential non-linear relationships, a quadratic polynomial (variable age) was added. Due to known age- and sex-differences [21, 22], formal tests for interactions with age (continuous) and sex were conducted by including interaction terms in the final regression model to assess potential effect modification. While no interaction with sex was observed, an interaction with age was observed and presented both in a continuous way (graphically) and in stratified analyses (stratification of age by quartiles Q1 37–49, Q2 50–57, Q3 58–62, Q4 63–73). Finally, in a sensitivity analysis, a multivariable Fine-Gray model was calculated to account for death as a competing risk for the occurrence of GERD.

The analysis was performed using IBM SPSS Statistics version 30.00.00.00, and R software version 4.4.0 (2024-04-24). A p-value of < 0.05 was defined as statistically significant.

## Results

### Descriptive analysis

Baseline characteristics of the 457,958 participants with and without incident GERD at follow-up are summarized in Table 1. The overall incidence rate of GERD in this population was 9.80%. The sample contained more women (57%) than men. Persons who developed GERD during follow up were older (median 60 years, IQR 53–64) than participants without GERD (median 57 years, IQR 50–63). Participants without incident GERD were physically more active. Individuals who developed GERD after baseline were less often current drinkers, more often current smokers, more often diagnosed with COPD, depression, and asthma at baseline. Individuals without GERD reported a significantly higher income and a higher level of education in comparison to participants with incident GERD.

**Table 1:** Baseline characteristics of patients with and without incident GERD. Categorial data is presented as total numbers (%). Numeric data is presented as median (IQR)

	Incident GERD *n* = 44,711 (9.80%)	No GERD *n* = 413,247 (90.20%)	*p*-value
Sex			< 0.001
- male	19,212(43%)	188,968(45.7%)	
- female	25,499 (57%)	224,279 (54.3%)	
Age (years)	60(53–64)	57 (50–63)	< 0.001
Alcohol consumption			< 0.001
- Never drinker	2,281 (5.1%)	17,236 (4.2%)	
- Ex drinker	2,033 (4.5%)	13,897 (3.4%)	
- Current drinker	40,345 (90.2%)	381,722 (92.4%)	
- Not reported	52 (0.1%)	392 (0.1%)	
Smoking			< 0.001
- Never-smoker	22,621 (50.6%)	229,939 (55.6%)	
- Ex-smoker	17,143 (38.3%)	139,729 (33.8%)	
- Current smoker	4,947 (11.1%)	43,579 (10.5%)	
Physical activity			< 0.001
- Low physical activity	6,752 (15.1%)	58,356 (14.1%)	
- Moderate physical activity	13,074 (29.2%)	131,606 (31.8%)	
- High physical activity	13,158 (29.4%)	132,188 (32.0%)	
- Not reported	11,727 (26.2%)	91,097 (22.0%)	
Depression			< 0.001
- Yes	5,129 (11.5%)	30,542 (7.4%)	
- No	39,582 (88.5%)	382,705 (92.6%)	
COPD			< 0.001
- Yes	1,258(2.8%)	6,122(1.5%)	
- No	43,453 (97.2%)	407,125 (98.5%)	
Asthma			< 0.001
- Yes	6,589 (14.7%)	43,257 (10.5%)	
- No	38,122 (85.3%)	369,990 (89.5%)	
Education			< 0.001
- A levels/ 0 levels	13,988 (31.3%)	134,975 (32.7%)	
- College/university	11,232 (25.1%)	140,883 (34.1%)	
- other	19,491 (43.6%)	137,389 (33.2%)	
Income			< 0.001
- More than 52.000 €	7,156 (16.0%)	96,301 (23.3%)	
- Between 31.000 and 52.000 €	9,004 (20.1%)	94,320 (22.8%)	
- Between 18.000 and 31.000 €-	10,163 (22.7%)	88,922 (21.5%)	
- Less than 18.000 €	10,785 (24.1%)	75,444 (18.3%)	
- Not reported	7,603 (17.0%)	58,260 (14.1%)	

*COPD* Chronic obstructive pulmonary disease

### Survival time analysis

**Fig. 1:**
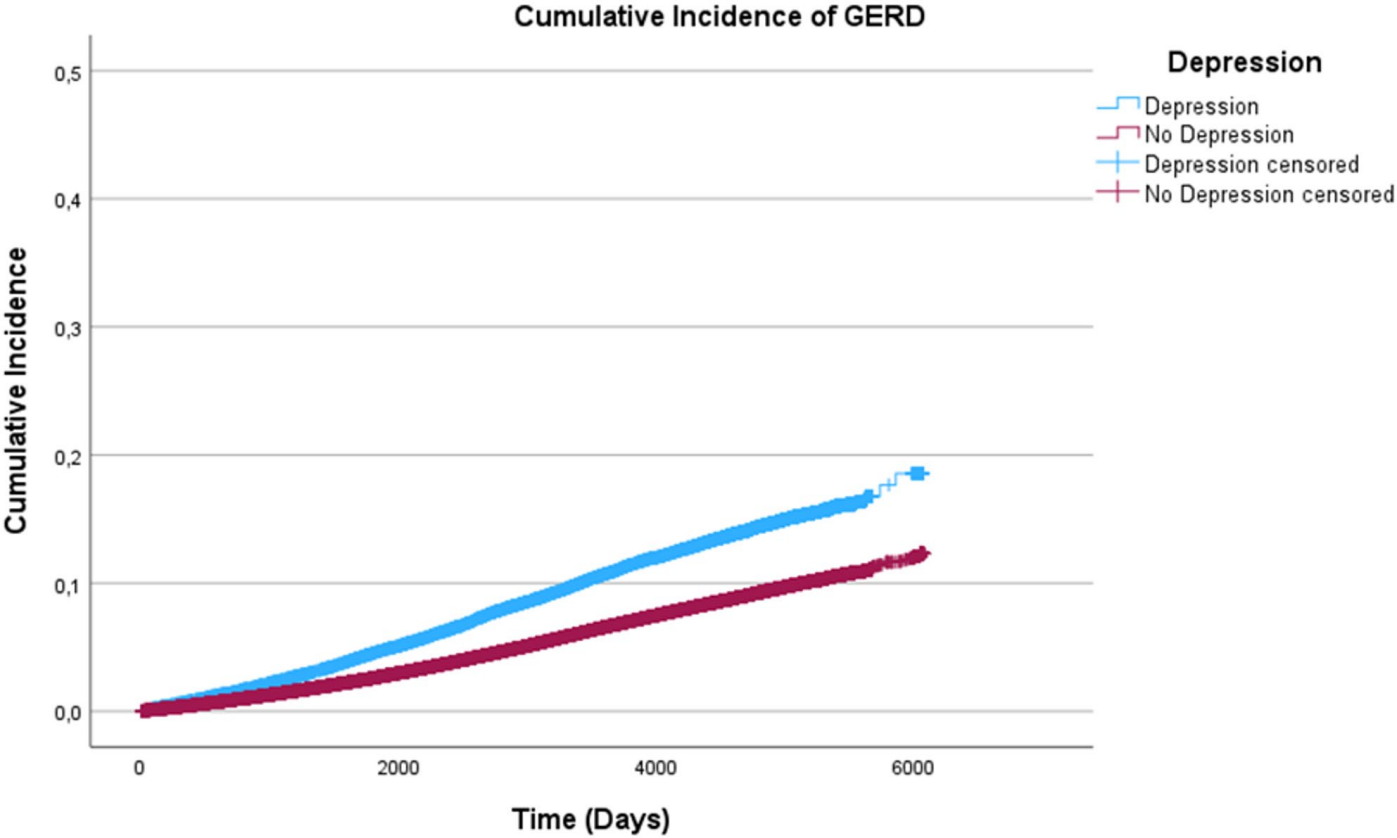
Cumulative incidence of GERD for participants with and without depression over time in days

Figure 1 displays the cumulative incidence of GERD for participants with and without depression over time. Participants with depression are more likely to develop GERD during follow-up (log-rank test: *p* = 0.001).

### Association between depression and GERD

The multivariable adjusted Cox regression model resulted in a significant association between depression and incident GERD (Hazard Ratio (HR): 1.51, 95% CI: [1.46,1.55]; *P* < 0.001). This result was fully supported by a similar estimate from competing risk analysis (considering 40,373 deaths) with an HR of 1.50 (95% CI: [1.46, 1.54]; *P* < 0.001).

**Fig. 2:**
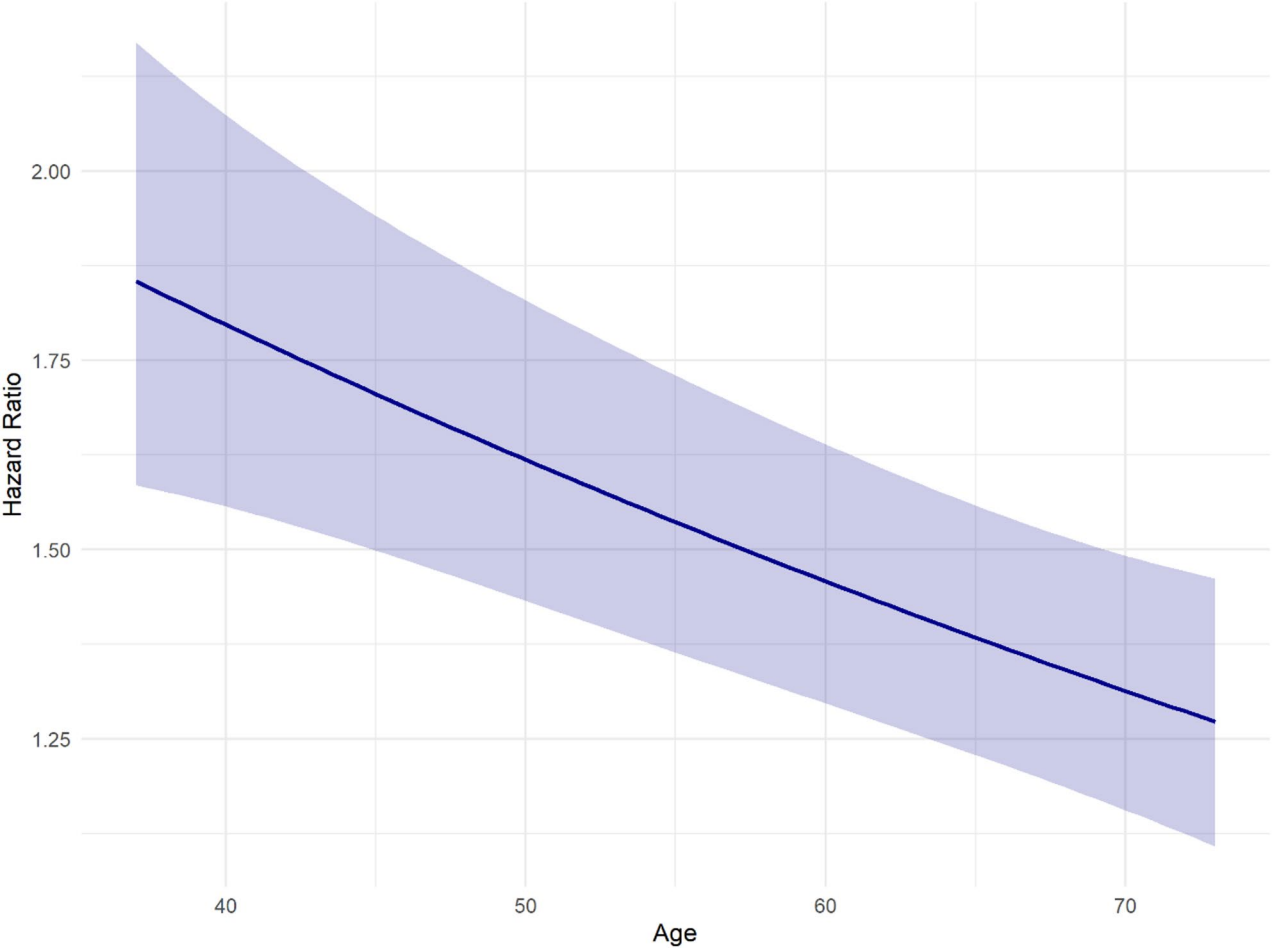
Association between depression and GERD depending on age, adjusted for sex, smoking, alcohol consumption, physical activity, chronic obstructive pulmonary disease (COPD), asthma, education and income

Due to the significant interaction with age, a multivariable Cox regression model including the interaction term was conducted. Fig. 2 illustrates the result of this analysis. It was found that the association between depression and incident GERD was stronger in younger participants. There was no significant interaction with sex.

This relationship was supported by the stratified analyses, where the strength of the association was highest in the youngest age group and decreased progressively across higher quartiles (Table 2).

**Table 2:** Association between depression and incident GERD stratified by age quartiles

Age quartile	Age range (years)	*N*	Hazard ratio (HR)	95% CI	*p*-value
Q1	37–49	112,086	1.61	1.51–1.72	< 0.001
Q2	50–57	119,775	1.60	1.52–1.70	< 0.001
Q3	58–62	105,377	1.44	1.36–1.53	< 0.001
Q4	63–73	128,209	1.37	1.30–1.46	< 0.001

Adjusted for sex, smoking, alcohol consumption, physical activity, chronic obstructive pulmonary disease (COPD), asthma, education and income

## Discussion

In the present study, depression at baseline examination was associated with the development of GERD (HR 1.51, 95% CI: [1.46–1.55]) over a median follow-up period of 13.52 (IQR 2.62–14.27) in 37 to 73 years old men and women from the UK. The association was stronger in younger participants and decreased with age.

A prospective cohort study based on data from the Health Improvement Network UK primary care database found a HR of 1.72 (95% CI, 1.60–1.85) [14] for developing GERD in patients with major depression, which is in line with a review article and two cross-sectional studies [23–25]. In a nested case-control study, with two different cohorts from Korea, the odds ratio (OR) for GERD in depressive patients was 2.01 (95% CI, 1.96–2.07) in the first cohort and 1.48 (95% CI, 1.43–1.52) in the second cohort [15]. A systematic review of Zamani et al., including particularly cross-sectional studies, strengthens the findings regarding a bidirectional association between anxiety/depression and GERD [13]. Finally, a bidirectional positive causal association between genetic liability of depression and GERD has been reported by a mendelian randomization study, with an OR for GERD of 1.51 (95% CI, 1.15–1.98; *p* = 0.003) [26]. The present study, based on a very large population-based sample, strengthens the previous evidence through the prospective design with a relatively long follow-up time.

### Pathophysiological mechanisms

Several psycho-physiological mechanisms may help to explain the link between depressive symptoms and the development of gastro-oesophageal reflux.

In patients with depression, the autonomic nervous system is dysregulated. The sympathetic nervous system is overactive, resulting in high stress levels, whereas the parasympathetic nervous system is underactive, slowing down the digestive process, resulting in pathophysiological dysfunctions, like reduced gastric accommodation, transient lower oesophageal sphincter relaxation (TLESR), and a change in gut motility [27–29]. It can also happen that stomach acid remains in the oesophagus for a longer time, causing discomfort [30]. A study in rats showed that an altered sensitivity to increased acid secretion leads to gastric damage in depressed animals [31]. This reinforces the assumption that acid regulation in the stomach changes under stress, through higher levels of gastric acid production and motoric through reduced closing ability of the lower oesophageal sphincter [32]. Depression and stress may cause a miscommunication in the interaction between the gut and the brain [33]. In addition, it is known that the gut and brain interact reciprocally, which may explain the bidirectional results of prior studies [34]. Reduced serotonin production can also affect the activity of the oesophageal sphincter and pain sensitivity. In one study, acute tryptophan depletion significantly lowered the threshold for pain sensitivity to chemical stimulation [35].

Several studies found increased levels of cytokines and chemokines, causing chronic peripheral inflammation in GERD patients [36, 37]. Miao et al. discussed that this could affect the central nervous system, causing a gradual development of depression, due to chronic inflammatory processes [26, 38–40]. As depression affects both the immune system and the inflammation rate [11], a vice versa relationship between chronic inflammation due to depression and gastric load resulting in GERD should be considered.

### Behavioural mechanisms

Behavioural patterns in individuals with depression, such as overeating, malnutrition, disordered sleep, psychomotor agitation and overall unhealthy lifestyle choices, like alcohol and tobacco consumption, can also affect the digestive system. Additionally, depressive patients might also have a higher sensitivity concerning reflux symptoms. Hypervigilance to external and internal factors, especially negative or alarming body signals, is a common symptom of depression and anxiety [13, 41, 42].

### Pharmacological mechanisms

The use of anti-depressant medication can strain the digestive system regarding GERD, especially through a dysregulated closing of the lower oesophageal sphincter [43]. One study reported that patients treated with tricyclic antidepressants were more likely to develop GERD, while SSRI were less likely [14]. However, a recent network meta-analysis found diverse gastric side effects of SSRIs, depending on the type of SSRI [12].

This study took the association between depression and GERD depending on age into account; the risk for GERD in depressive patients decreased with increasing age. We hypothesize that this may be due to the presence of other, more severe comorbidities which increase with age, potentially overshadowing reflux symptoms and the diagnosis of GERD.

Older people more frequently take medications, such as cardiovascular medication, painkillers or proton pump inhibitors [2]. These could also mitigate or influence symptoms of reflux. Prevalence of GERD is not decreasing with age, but the way it is perceived can change [44]. In addition, the sensitivity to pain in the oesophagus can decrease with increasing age [10]. A natural decline of serotonin with age might also weaken the effect of serotonin deficiency in depression [45]. Multiple psychophysiological changes with age, e.g. hormonal, metabolic, or emotional changes as well as lifestyle modifications, should also be considered [46–48].

### Strengths and limitations

The strength of this study is the very large population-based sample of more than half a million participants that ensured a high statistical power. Furthermore, the longitudinal design allows exploring the association between depression and the incidence rather than just the prevalence of GERD. The renowned dataset was obtained through standardised data collection and fulfils high-quality criteria. The broad data collection together with the available sample size allowed an extensive adjustment in the statistical analyses.

Several limitations should be noted. No adjustment was made for confounders like sleep behaviour, eating habits, and Helicobacter pylori status (the latter because H. pylori status was available only for a subsample of the UK Biobank). We also did not adjust for the use of antidepressants. However, it cannot be ruled out that the observed association is partly attributable to treatment with antidepressants and not only (or additionally) to depression itself. Some variables had missing data which led to a slightly reduced sample size in fully adjusted regression models. Disease diagnoses in the UK Biobank are derived from multiple sources within the UK healthcare system, enhancing comprehensiveness and representativeness; however, reliance on self-reported symptoms and diagnostic codes may introduce heterogeneity and bias. Depression was assessed at a single time point, which likely led to non-differential misclassification of exposure (some participants with past or intermittent depression were classified as unexposed). Such misclassification typically biases associations toward the null, underestimating the true effect. Additionally, a single assessment cannot capture the fluctuating or time-varying nature of depression, which may obscure important temporal patterns (e.g., stronger associations shortly after an episode that will weaken over time).

Self-report for GERD is a significant source of potential bias, as symptoms are common and self-interpretation varies. The inclusive GERD case definition used in this analysis may have caused non-differential misclassification by including individuals with mild or transient symptoms while excluding those with atypical presentations not reported at assessment, again biasing results toward the null and possibly reducing our ability to detect associations in clinically significant GERD.

Individuals with depression tend to utilize healthcare services more frequently, leading to higher detection and recording of GERD (surveillance/detection bias). This could artificially inflate the observed association. Since the UK Biobank is a volunteer cohort with an initial age range of 37 to 73 years, the results are only partially transferable to settings with poorer access, other age groups, or different cultural/healthcare system contexts. Furthermore, the hypotheses (comorbidities eclipsing symptoms, changing perception of symptoms, medication use) regarding the age interaction could not be tested with the current data. Future research is necessary to confirm our findings and to investigate possible underlying mechanisms.

## Conclusion

In the general UK population, depression is associated with an increased risk of GERD. The relationship decreases with age. The potential need for specific screening measures or prevention strategies regarding the development of GERD for patients with depression should be considered by the treating physicians.

## Supplementary Information

Supplementary Material 1.

## Data Availability

The datasets used and/or analyzed during the current study are available from the UK Biobank under approved project application number 192645, but restrictions apply to their availability. Access to the UK Biobank resource is granted to bona fide researchers through a formal application process, which requires institutional affiliation and payment of access fees. Researchers interested in accessing the data can apply via the UK Biobank website ( [Use our data - UK Biobank] (https://www.ukbiobank.ac.uk/use-our-data) ). The authors do not have permission to share the data directly. Data are, however, available from the authors upon reasonable request and with permission of the UK Biobank.

## Abbreviations

Abbreviation: Definition
COPD: Chronic Obstructive Pulmonary Disease
GERD: Gastroesophageal Reflux Disease
HR: Hazard Ratio
ICD: International Classification of Diseases
IQR: Interquartile Range
MREC: Medical Research Ethics Committee
SSRI: Selective Serotonin Reuptake Inhibitor
TLESR: Transient Lower Esophageal Sphincter Relaxation
