# Supplementary material for: Depression and risk of gastro-oesophageal reflux disease (GERD): results from the UK Biobank study
Source: BMC Gastroenterol. 2026 Jan 8;26:67. doi: 10.1186/s12876-025-04591-7 (PMC12837071; doi:10.1186/s12876-025-04591-7)
Supplement: Supplementary file 1 — Supplementary Material 1. [file 12876_2025_4591_MOESM1_ESM.docx]

***Supplementary Material***

***Title:*** Depression and risk of gastro-oesophageal reflux disease (GERD): Results from the UK Biobank study

***Figure S1:*** Flowchart of all included and excluded cases


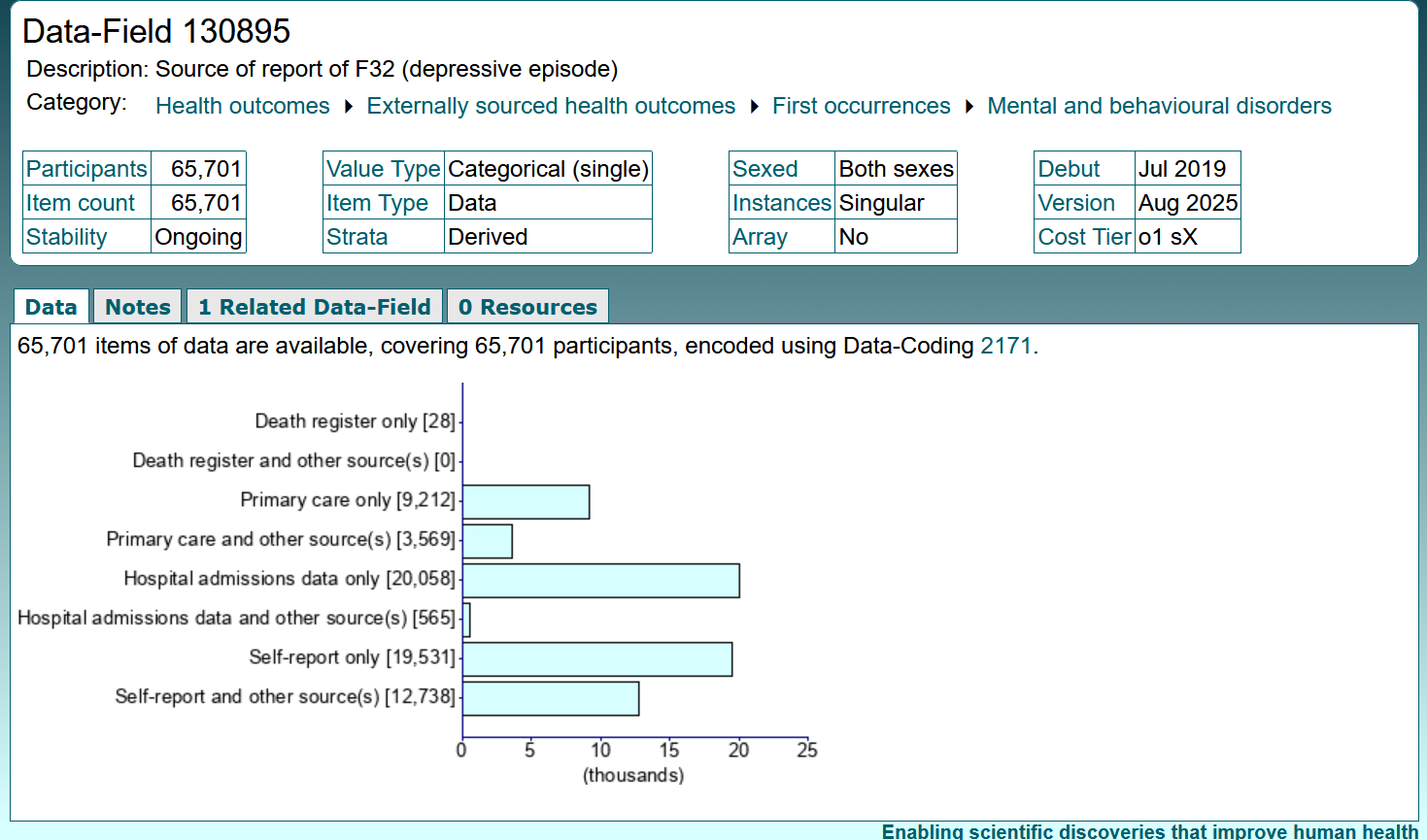


***Figure S2:*** Distribution of baseline cases of ICD F32 across data sources


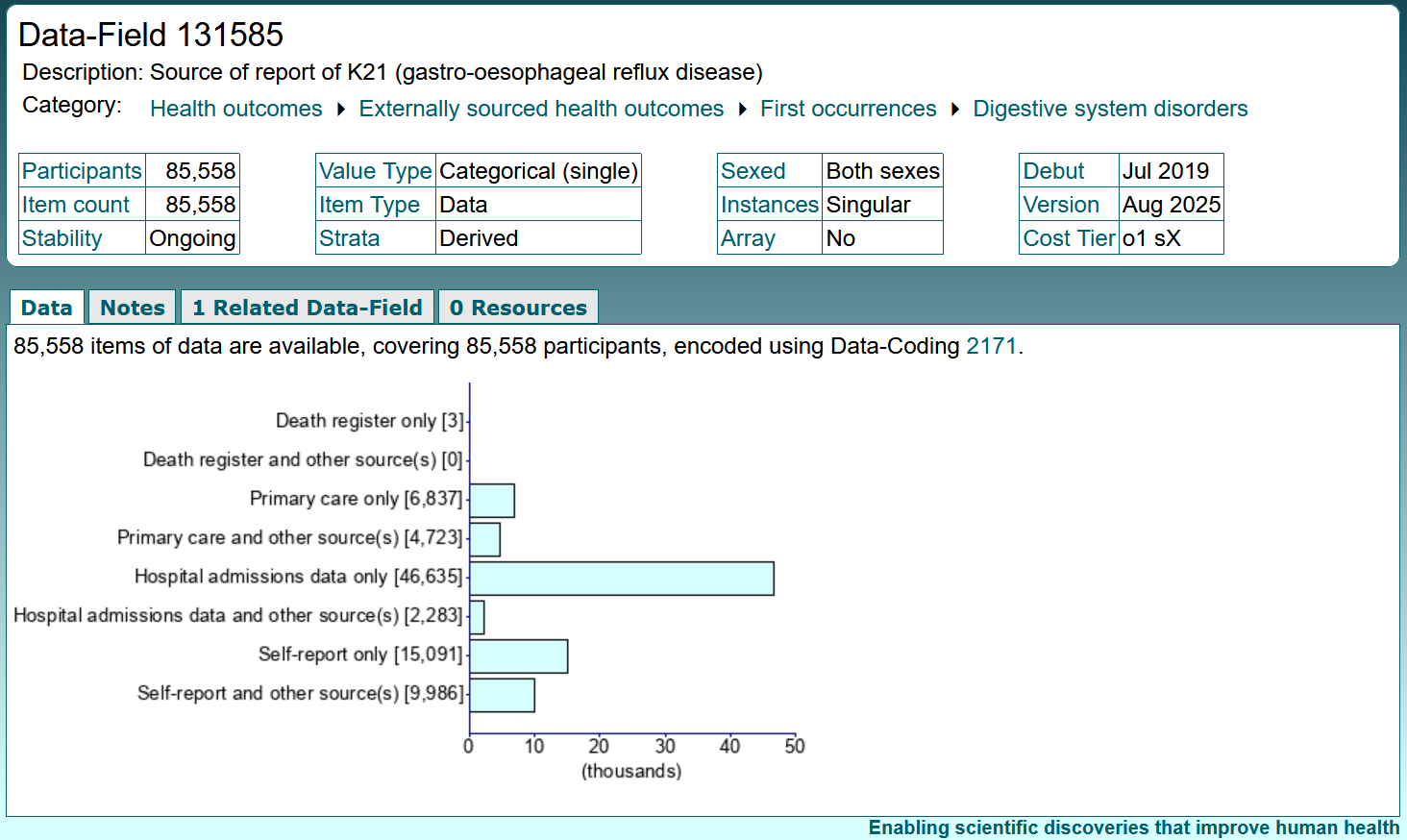


***Figure S3:*** Distribution of baseline cases of ICD K21 across data sources


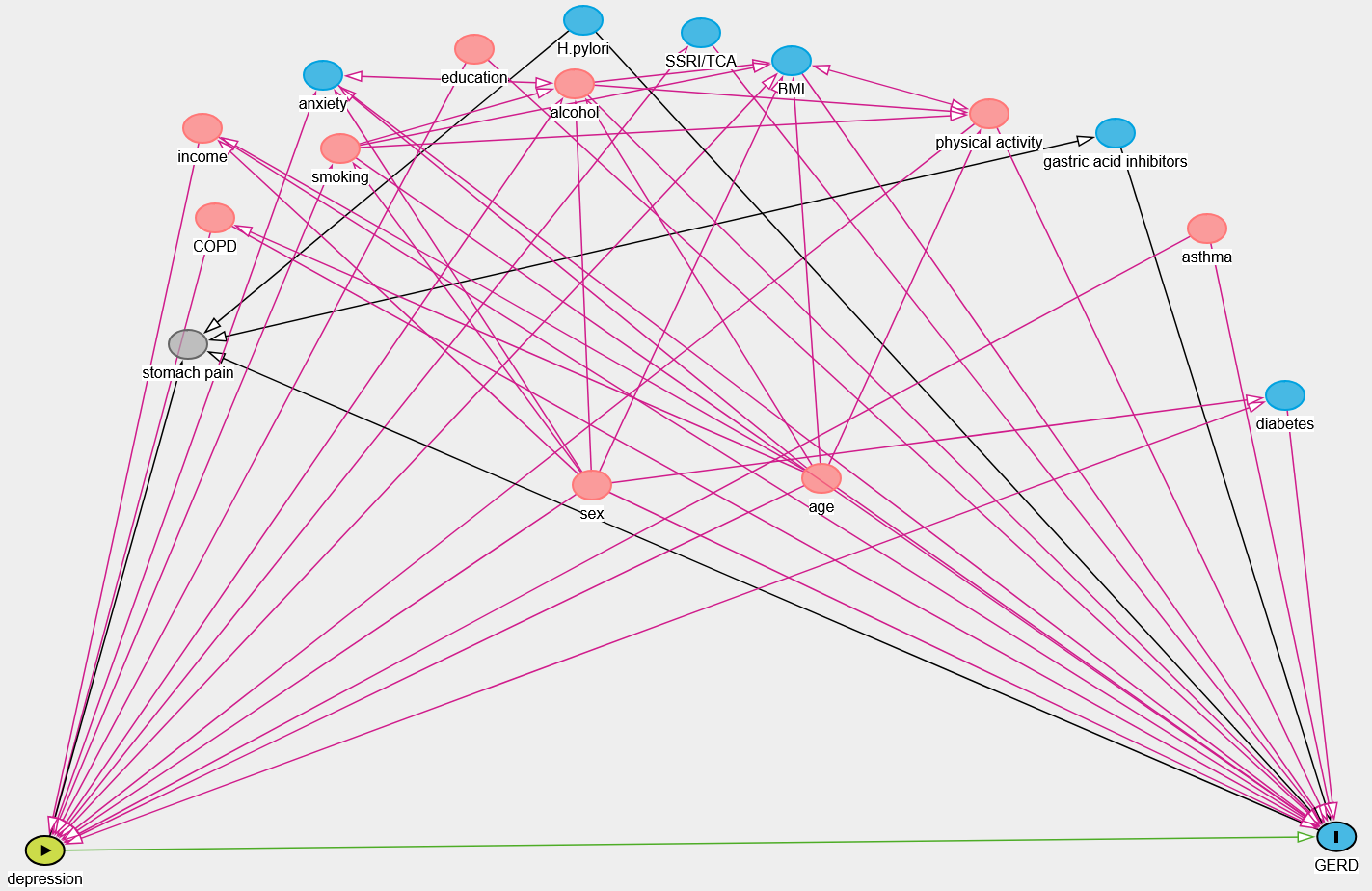


***Figure S4:*** Directed Acrylic Grap (DAG) with selection of covariables
